# Preliminary Education and College Training

**Published:** 1903-02-15

**Authors:** Maurice Roy


### PRELIMINARY EDUCATION AND COLLEGE TRAINING.

BY DR. MAURICE ROY.

Extract of Report to the International Dental Federation, at its Meeting in Stockholm.

*First question*—"What are the preliminary studies to be required of students before beginning their professional education?"

The necessity of a sufficient general education is admitted by everyone and I think it needless to insist thereon. What we have more particularly to consider is, (1) The extent of such general education; and (2) the age at which it should finish.

These two considerations are in reality absolutely inseparable. For if the question of age had not to be considered, there would be no reason why one should limit the general education of the dentist, the general culture of a man being never too great, no matter to what branch of human industry he may be called. But for the dentist a limiting consideration here interposes—that is, the neceessity of his beginning at an early period of life the study of his profession properly so called.

For these reasons we think that the studies relating to general culture ought not to be prolonged beyond the age of sixteen; not that we think it necessary to commence dental studies at thet age, but because we consider that the education of the future dentist should then assume a special character in order to prepare him progressively for dental studies properly so called.

What is to be the program of these studies of general culture? It will evidently be variable according to the country and the educational organization; it ought nevertheless to form a complete whole, quite homogeneous, allowing a youth of sixteen to have a general idea of the fundamental bases of human knowledge.

This being recognized as regards general culture, are the studies of the future student of dentistry to be confined solely to this general education, or are they to be already directed to the special object he has to pursue?

In order to solve this question, we must first put another, on the solution of which will depend the answer of the first: What qualifications are necessary to make a dentist? Everybody has not the aptitudes necessary for a dentist, who should possess, besides an intelligence sufficient to assimilate the varied knowledge necessary for the study of our art, enough manual skill to acquire in that time the dexterity required in the execution of the diversified labors of operative dentistry and prosthesis.

Now, there are certain individuals who, though endowed with intelligence in every other respect, are naturally so awkward as to render them incapable of acquiring even medium skill in the practice of our art. It appears therefore necessary, according to the principle laid down by Mr. Cunningham, to make sure beforehand that the future student of dentistry possesses this indispensable minimum of manual skill. For this reason it would be advisable to require from the latter a general manual instruction that would enable one to form an opinion as to his aptitude and at the same time would cause him to acquire, from this point of view, the general principles the application of which he would meet with later on. It is also advisable that before entering upon his professional instructions the student of dentistry should possess general scientific notions sufficient to follow profitably the special courses of lectures, or otherwise these would have to be kept at too elementary a level.

It is to these two orders of instruction—gradual manual instruction on the one hand, general and elementary scientific instruction on the other—that the preliminary studies of the student of dentistry must be devoted from sixteen to eighteen, the normal age of admission to the school of dentistry. To these must be added the study of two living languages, the necessity of which has been felt at meetings like these we hold annualy.

These different considerations have led us to propose to you to adopt, for the first question put by the International Dental Federation, the following answer, which is the reproduction of a wish expressed by the Congress of 1900:

The *preliminary education necessary* for the student of dentistry before being admitted to follow his professional education must comprise—

(1) A literary education with a knowledge of two living languages.

(2) An elementary knowledge of sciences.

(3) Manual instruction.

On these different orders of instruction we cannot seek for better inspiration than in the remarkable words of Sir Michael Foster when he speaks of the general basis on which all special education must rest. The broad basis, he says, must be "the discipline of the school; that is, the boy's school. I say the discipline rather than the learning of the school, for the aim of the schoolmaster should be in all cases the formation of the mind—the setting of the instrument, not the filling of the bottle. The growths of habits of accuracy, of intentness, and of alertness, this rather than the gathering of mere knowledge of facts is the proper heritage of the school, and for the attainments of these habits it matters not so much what the boy is taught as how he is taught.

*Second question*—"In what are dental studies to consist, how long ought they to last, and what should be the order of the program?"

It is incontestable that a man's education is never too extensive; doubtless all knowwledge is useful, but, as Sir Michael Foster in his address at Cambridge admirably remarks, "The power of the human mind to acquire and preserve knowledge is limited. As Dr. Godon remarks, quoting Herbert Spencer, 'We that have but span-long lives' must ever bear in mind our limited time for acquisition."

The question of how long studies ought to last is therefore of extreme importance. Their duration must be perforce limited and four or five years seems to be a maximum that can not be exceeded. It would be impossible to require from dentists studies analogous to those of doctors of medicine, since it is impossible, as no one sufficiently acquainted with the facts will deny, in four or five years to make at once a doctor and a dentist. It is advisable therefore to make a rational use of the maximum time to be devoted to study, so as to allow students of dentistry to acquire gradually the knowledge requisite for the pratice of their profession.

Dental studies come under two heads: on the one hand scientific and medical studies, on the other technical studies. How are they to be divided?

We consider it indispensable to give a very notable pre-eminence to technical studies, and particularly to practical work. Dental therapeutics and its two corollaries, operative dentistry and prosthesis, admit of a very important manual part, necessitating along apprenticeship both of the eye and hand, hence the necessity of devoting a considerable part of the hours allotted to study to practical work (prosthesis and operative dentistry).

Is it desirable to divide the teaching as is done in certain foreign countries—Switzerland, for instance, where one year is devoted to the study of physical and natural sciences, one year to medical studies, and a year and a half to dental studies? We are not of that opinion. We have already said that technical studies should be of long duration, particu'arly as regards practical work; now, in order that the student may profit by the latter, it is necessary that he should receive concurrently theoretical technical instruction without prejudice to general scientific and medical instruction. From the above facts the necessity follows of organizing different courses of lectures so that medical and scientific instruction and technical instruction may be given simultaneously. The distributional one should differ; scientific and medical lectures should predominate in the first years and technical lectures in the last. All the courses of lectures, especially the technical ones, should naturely be graduated according to the years of study.

It is desirable that a particular regulation should be made applying to doctors of medicine who intend to practice dentistry; and conformably to the wish expressed by the Congress of 1900, it should be obligatory for this category of students to attend for two years the classes of a school of dentistry. It would, however, be necessary to draw up a program of practical work for these students so as to allow of their going gradually through the program of practical work which normally exends over four years.

These diverse considerations being settled, we propose to you to adopt the following answer to the second question above stated:

(1) Dental studies should comprise a scientific and medical part, and a technical part.

(2) Their duration must be at least four years.

(3) These studies must be organized conformably to a parallel method for all the classes—scientific and medical instruction and technical instruction simultaneously.

(4) It must be obligatory on medical graduates who propose practicing the art of dentistry to attend, for at least two years, the lectures of a school of dentistry.

*Third question*—"What branch of the studies, as taught in medical schools, must the student of dentistry follow?"

After what we have just said on the preceding question, the answer to this last question does not admit of being extended. In the first place we reject completely, for reasons that have been often stated and which a few are here recalled, the assimilation of dental studies to medical studies and consequently the obligation of completed medical studies in order to practice the art of dentistry.

Irrespective of this absolute view of the question, is there an advantage for students of dentistry to follow certain teachings given in schools of medicine? We think not. As we have already said if it be indispensable that the student of dentistry should have a knowledge of anatomy, physiology, chemistry and physics, it is not indispensable that his knowledge of these subjects should be as extensive as if he were destined to practice medicine or to take a degree in physical sciences. In the faculties of medicine lectures are given with a view to medical studies; it follows therefore that students of dentistry having but a limited time to devote to them can profit only to a limited extent of lectures of a higher order than is strictly necessary for their later studies, or else, should they devote sufficient time to them, unless their studies be prolonged it must be to the detriment of their professional instruction.

The conclusion we arrive at is that if dental science is an autonomous science then dental instruction must be autonomous instruction.

There remain, however, two particular points to be examined: dissection on the one hand, medical and surgical clinics on the other.

As regards dissection, dental schools have a difficulty to contend with, namely, that of procuring corpses, many considerations interposing in many countries with respect to this. In countries where this difficulty exists it is absolutely necessary to have recourse to medical schools or to hospitals, but subject to one peremptory condition, namely, that lectures shall be given there exclusively for the use of dentists, the *locale* alone being changed, the spirit of the teaching remaining the same..

With respect to medical and surgical clinics the question is easier to solve. We must bear in mind that with very rare exceptions—and then they are generally assisted by the doctor in attendance—dentists have only to attend patients who are not confined to their beds. Consequently what they have an interest in seeing patients coming and going like those who present themselves at the consultations for out-door patients. It is therefore easy to have a consultation room annexed to each school, in order to supply this want; but, in default of such a plan, it will be necessary to apply to the hospitals, bearing in mind, as for dissection, that the examination of patients be made strictly with a view to the special teaching to be followed by students of dentistry.

We therefore propose to you, in answer to the third question, to adopt the following resolution:

"All the studies of students of dentistry must take place exclusively in schools of dentistry, except that of dissection in cases where it is impossible to procure dead bodies, but subject to the condition that the lectures be given exclusively for the use of such students."—*Dental Cosmos*.

